# Effects of Organizational Support and Organizational Justice on Police Officers’ Work Engagement

**DOI:** 10.3389/fpsyg.2021.642155

**Published:** 2021-07-21

**Authors:** Andrzej Piotrowski, Samir Rawat, Ole Boe

**Affiliations:** ^1^Faculty of Social Sciences, Institute of Psychology, University of Gdańsk, Gdańsk, Poland; ^2^Military MIND Academy, Pune, India; ^3^USN School of Business, Department of Industrial Economics, Strategy and Political Science, University of South-Eastern Norway, Drammen, Norway

**Keywords:** work engagement, organizational support, organizational justice, police officers, supervisor support, supervisor justice

## Abstract

The impact of organizational support and organizational justice on work engagement was investigated in a group of police officers. A review of the literature revealed that studies reporting differences between the influence of supervisors and coworker justice and support on work engagement among police officers are grossly insufficient. This study hypothesized that organizational support and organizational justice would positively predict work engagement among police officers. It was also hypothesized that, among police officers, supervisor support is more strongly related to work engagement than coworker support and that supervisor justice is more strongly linked to work engagement than coworker justice. Participants were 170 police officers who worked in police departments in northern Poland. A regression analysis showed that supervisor support and supervisor justice had a positive effect on police officers’ work engagement, whereby organizational support coupled with organizational justice accounted for 26% of the variability of work engagement. Theoretical and practical implications are discussed, and directions for future research are suggested.

## Introduction

In organizational psychology, work engagement (WE) has been defined as “a positive, fulfilling, work-related state of mind that is characterized by vigor, dedication, and absorption” (Schaufeli et al., 2002, p. 74). The concept of WE by Schaufeli et al., which has most often been explored in the context of human resource management, using the Utrecht Work Engagement Scale (UWES), has been implemented in more than 30 countries and across various professions (e.g., Petrović et al., 2017; Carmona-Halty et al., 2019; Lazauskaitė-Zabielskė et al., 2020). Perceived organizational support (POS) is an important psychological resource for work resources. POS is a subjective feeling that an organization cares about employee involvement in work and well-being (Zeng et al., 2020). POS addresses employees’ perceptions about the extent to which their organization highly values their contribution and promotes their welfare (Eisenberger et al., 1986). Empirical evidence shows that POS is associated with a number of positive organizational consequences that have been categorized into three main categories of outcomes, i.e., (1) subjective employee well-being, (2) positive employee attitudes toward organization and work, and (3) favorable employee behavior (Caesens and Stinglhamber, 2020). Organizational justice refers to employee’s perception of fairness (Pan et al., 2018). Distributive justice refers to the belief that resources that are allocated to people are “deserved” or not, depending on their contribution. Procedural justice refers to the fairness of the means by which payments are made or decided upon. Interactive justice refers to the respectful and correct way in which authorities communicate the details of procedures and justify their decisions using honest and truthful information (Correia and Almeida, 2020).

The Police is a uniformed and armed formation designed to protect the safety of people and property, and to maintain public safety and order. Its main tasks include ensuring compliance with the law and prosecuting criminals, as well as providing protection and assistance in crisis situations, both for people and property (Letkiewicz and Majer, 2016).

The main aim of the article is to explore how the organizational justice and organizational support coming from supervisors and co-workers affect work engagement among police officers.

In 1987, Greenberg introduced the concept of organizational justice with regard to how an employee judges the behavior of the organization and the employee’s resulting attitude and behavior (Greenberg, 1987). Organizational justice (OJ) is a concept composed of the following dimensions: distributive justice (DJ), procedural justice (PJ), retributive justice (RJ), interactional justice coming from supervisors, and interactional justice coming from coworkers (Macko, 2009). In organizations that are perceived as being concerned with the welfare of their employees, the environment could be thought of as one characterized by organizational support (OS) (George et al., 1993; Fasolo, 1995; Shore and Shore, 1995). Both OJ and OS can be viewed as organizational determinants with a possible impact upon workers WE. The main research question in this article is which of the dimensions of OJ and OS have the greatest relationship to WE among police officers.

## Literature Review

According to the Job Demands-Resources (JD-R) model, the main sources of WE are resources in one’s workplace (Bakker and Demerouti, 2007). The authors claim that work can be characterized by various social, psychological, and physical difficulties that can be described as work requirements (e.g., work overload, workplace conflicts). At the same time, work creates opportunities and possibilities that constitute work resources (e.g., autonomy, being able to learn, training opportunities and equipment); these resources are also properties manifested by the worker (e.g., self-efficacy, optimism). According to this model, WE develops if the resources of a workplace and an employee are sufficient; however, if resources are low and demands are high, however, emotional exhaustion can appear, followed by occupational burnout. Schaufeli and Bakker (2004) point out that for engagement to develop, work resources must match the demands of the work required. On the other hand, *occupational burnout* occurs a lack of resources at work is coupled with high demands (Schaufeli and Bakker, 2004).

Burnout can result from increasing work requirements, such as emotional demands, work overload, and work family life overlap, and decreasing work resources, such as social support, autonomy, possibilities to learn, and feedback (Schaufeli et al., 2009). Increasing burnout predicts a reduction in WE. A meta-analysis of studies within the JD-R model have shown that work resources such as social support, autonomy, feedback, positive organizational climate, and a sense of self-efficacy are significantly and positively linked to WE (Halbesleben, 2010). Furthermore, a meta-analysis by Nahrgang et al. (2011) showed that professional burnout is positively correlated with work that requires higher levels of danger, risk, and job complexity, whereas resources such as coworker support, manager support, knowledge, professional autonomy, and a sense of security at work are linked to engagement. Schaufeli et al., 2009, studying police officers and using the theory of perceived organizational support (POS), conservation of resource (COR), and job demands-resources (JD-R) model, found that POS not only directly affected police job burnout but also indirectly affected police job burnout through job satisfaction. Studying Polish military officers, Piotrowski et al. (2020a) found that organizational support and climate were important for citizenship behaviors in the army. Drawing commanders’ and superiors’ attention to these aspects of the army functioning may help them to shape and promote citizenship behaviors in a better way.

Many previous researchers have found a number of personal and organizational determinants of WE. For instance, Langelaan et al. (2006) analyzed the relationship between personality and temperament with WE, and showed that employees with high levels of WE are characterized by low neuroticism in combination with high extroversion and high levels of mobility. Regrettably, the study was only correlational and did not study the influence of other personality traits, such as conscientiousness. According to Li et al. (2014), a proactive personality moderated the effects of social support on WE. However, it is difficult to generalize these results due to the study’s cross-sectional design and the fact that regional differences were not considered in the analysis.

Further research has reported that WE correlates strongly with positive affectivity (0.65) and positive mood (0.35), but the causality of this relationship has not been studied, and reciprocal and dynamic linkages between mood, WE, and goal-directed behavior have not been determined (Bledow et al., 2011). Adaptivity, emotional maturity, positive attitudes, and results orientation have also been identified as predictors of WE (Langelaan et al., 2006). Xanthopoulou et al. (2013) showed the positive impact of self-efficacy, personal resources, and optimism on WE. Furthermore, according to Mostert and Rothmann (2006), conscientiousness and extroversion may predict vigor and dedication in police officers. Finally, the role of WE in integrating police wellness and ethics has been investigated by Blumberg et al. (2020).

Unfortunately, most of these previous studies are based on self-reports and were carried out in single organizations, which limits the interpretation and generalization of the results. This is particularly important as police forces differ significantly from civil institutions (Swid, 2014).

The principles and managing styles found within this organization specifically determine the staff’s organizational behavior. So far, no research on organizational determinants of WE among police forces has been conducted. Police officers constitute a large occupational group in almost every country, yet the number of studies on WE that take police officers into account is insufficient.

OS (Lyu, 2016), OJ (Shantz et al., 2016), and inclusive leadership (Mäkikangas, 2018) have been classified as organizational determinants. WE has a positive impact on many organizational behaviors. Namely, a high level of WE has been linked to low absence, low employee turnover, low levels of occupational stress, and high job satisfaction (Austin et al., 2020). WE also affects productivity and organizational citizenship behavior (Kataria et al., 2019). Engaged employees are characterized by higher productivity and organizational citizenship behaviors. This is of particular importance since the occupation of police officers involves many organizational demands.

Specific working conditions of police officers can lead to occupational stress (e.g., through contacts with criminals and violence, having to use physical force or even kill an assailant attacking an officer) (Pastwa-Wojciechowska and Piotrowski, 2016). The police, as one of the organizations responsible for safety, implement modern management methods. OS (Gillet et al., 2013) and leadership (Breevaart et al., 2014) have been identified as determinants of WE among police officers.

Research conducted by Basinska and Dåderman (2019) among policemen found that job burnout is negatively related to the cognitive intrinsic values of work (Challenge, Variety and Creativity, while work engagement is positively associated with a group of intrinsic work values (Challenge, Variety, Altruism, Creativity, and Achievement), as well as with external work values (Associates and Prestige). The particular values displayed by policemen may predispose them to professional burnout or work engagement.

One study carried out among soldiers showed that OS from superiors is lower than OS from other soldiers (Stetz et al., 2006). Superior support is a vital work resource in the job of a police officer. Job satisfaction mediates a positive relationship between OS and working among policemen (Lan et al., 2020). Furthermore, Liu et al. (2019) reported that WE affected the police officer’s life satisfaction through work-family conflict. Most studies, however, fail to distinguish between OS coming from one’s superiors and OS coming from coworkers. This differentiation is critical because of the many work-related demands of police officers and the authoritarian leadership style used within police forces, which is characterized by little support for subordinates (Fenici et al., 2011). Earlier researchers have shown that the individual dimensions of OJ (PJ, DJ, and IJ) are related to WE to a similar extent (measured by the correlation factor Pearson’s r) which for procedural justice, PJ = 0.56; for distributive justice, DJ = 0.59; and interactional justice, IJ = 0.57) (Lyu, 2016). Research from Canadian police organizations suggests that when police officers felt they had been treated fairly, they felt a greater sense of psychological security, which in turn improved their identification with the organization and increased their work engagement (Gelderen and Bik, 2016).

According to Macko (2009), OJ is composed of the following dimensions: distributive justice (DJ), procedural justice (PJ), retributive justice (RJ), interactional justice coming from supervisors, and interactional justice coming from coworkers. *Distributive justice (DJ)* is high, when the profits from work are distributed in proportion to each coworker’s individual contribution. *Procedural justice (PJ)* is determined by the extent to which the coworkers may present their own views and influence organizational decisions, ensuring equal representation of all parties and fair appeal procedures (used to change bad decisions). *Retributive justice (RJ)* is determined by the extent to which punishments are consistent with common moral standards and resistant to any individual influence. *Interactional justice coming from supervisors* means providing clear and adequate explanation of the decision-making process and its results, as well as supervisors’ communication being honest, open, and free from any misrepresentations or attempts at manipulation. *Interactional justice coming from coworkers* entails respecting the dignity of other coworkers and treating them in an unbiased way without interfering with their privacy.

Given an authoritarian leadership style is used in the Polish police force, we believed it would be interesting to investigate how the support of supervisors and coworkers as dimensions of OS, as well as the dimensions of OJ such as distributive justice (DJ), procedural justice (PJ), retributive justice (RJ), interactional justice coming from supervisors, and interactional justice coming from coworkers are linked to WE. Stetz et al. (2006) who conducted their research among soldiers, it can be assumed that police officers’ support from coworkers will be greater than the support they receive from supervisors. Thus, we proposed the following hypotheses:

**Hypothesis 1:** Supervisor support will be more strongly related to police officers’ work engagement than will support from coworkers.
**Hypothesis 2:** Supervisor justice will be more strongly related with police officers’ work engagement than will coworker justice.

## Methodology

This quantitative study was carried out in accordance with the recommendations of the APA and Code of Ethics and Professional Psychologist of the Polish Psychological Association (PTP) (Knapp et al., 2012; Brzeziński, 2016). No identifying information was placed at the questionnaires. All subjects gave written informed consent in accordance with the Declaration of Helsinki World Medical Association [WMA] (2013). The presented study is part of a research project that aims to determine key organizational variables affecting WE in uniformed services (armed forces, police, and prison service officers). OJ was measured with the Organizational Justice Questionnaire (Macko, 2009), while OS was measured using the Social Support subscale derived from the Psychosocial Working Conditions Questionnaire (Widerszal-Bazyl and Cieślak, 2000), and WE was measured by using a Polish adaptation (Szabowska-Walaszczyk et al., 2011) of the Utrecht Work Engagement Scale (UWES) developed by Schaufeli et al. (2002).

### Participants and Procedure

Participants were 170 police officers (42 women, 128 men) including 25 supervisors (five women, 20 men), with a mean age of 32.32 years (*SD* = 6.84), all of whom worked in police departments in northern Poland. The policemen came from various departments: Criminal 28.0%, Prevention 45.1%, Road 14.3%, Logistical 0.5%, Special Forces 10.4%, Judicial Police 1.6%. Police officers working in shifts made up 85.6%. Command positions were occupied by 16.1%. Secondary education was 1%, Bachelor’s level 40% and Master’s level 39%. Most of them were in close relationships (marriage 59.9% or cohabitation 11%), only 10% was single.

A convenience sample was used in the study. Participation in the study was voluntary, and the study itself took place at the end of the shift. Additionally, on the first page of the survey, we stipulated that the respondents’ answers would be used only for research purposes. The group included also supervisors who were completing questionnaires during the briefing in a different room than their subordinates. All respondents were informed that their answers would be treated as confidential. To ensure anonymity and comfort in responding, sets of questionnaires were delivered in envelopes. After answering the questions, the respondents inserted the envelopes into an opaque box.

### Variables

#### Organizational Support

OS was measured using the Social Support subscale derived from the Psychosocial Working Conditions Questionnaire (Widerszal-Bazyl and Cieślak, 2000). The subscale consists of 16 items; half of the items measure support from the coworkers (Cronbach’s alpha, αCR = 0.91), and half assess support from supervisors (αCR = 0.92). An example of the item of the support from the coworkers scale: “To what extent can you count on getting helpful, everyday information you need from your colleagues?” An example of the item of the support from the supervisors scale: “To what extent can you count on your superiors helping you in some particular way?” Participants were asked to indicate on a 4-point Likert scale ranging from 1 (very small) to 4 (very large), describing to what extent they agreed with the statement.

#### Organizational Justice

OJ was measured using the Organizational Justice Questionnaire (Macko, 2009), which consists of 30 items divided across the following subscales: distributive justice (DJ) (αCR = 0.91; 7 items, e.g., “The amount of my remuneration corresponds with the amount of my responsibility”), procedural justice (PJ) (αCR = 0.83; 7 items, e.g., “Coworkers have a guaranteed influence on decisions made”), retributive justice (RJ) (αCR = 0.73; 4 items, e.g., “Coworkers are not charged without a reason”), interactional justice coming from supervisors (αCR = 0.88; 8 items, e.g., “What supervisors say is consistent with what they do”), and interactional justice coming from coworkers (αCR = 0.73; 4 items, e.g., “My coworkers treat me with respect”). Responses were made on a 4-point Likert scale ranging from 1 (I definitely disagree) to 4 (I definitely agree).

#### Work Engagement

WE was measured using the Utrecht Work Engagement Scale (UWES) (Schaufeli et al., 2002) in a Polish adaptation (Szabowska-Walaszczyk et al., 2011), which comprises 17 items, six of which are used to assess vigor (αCR = 0.80; e.g., “At work, I feel like I am bursting with energy”), five to assess dedication (αCR = 0.91; e.g., “My job inspires me”), and six to assess absorption (αCR = 0.75; e.g., “I am immersed in my work”). αCR for the whole scale was 0.93. Responses were made on a 7-point Likert scale ranging from 0 (never) to 6 (every day).

##### Control Variables

**FIGURE 1:**
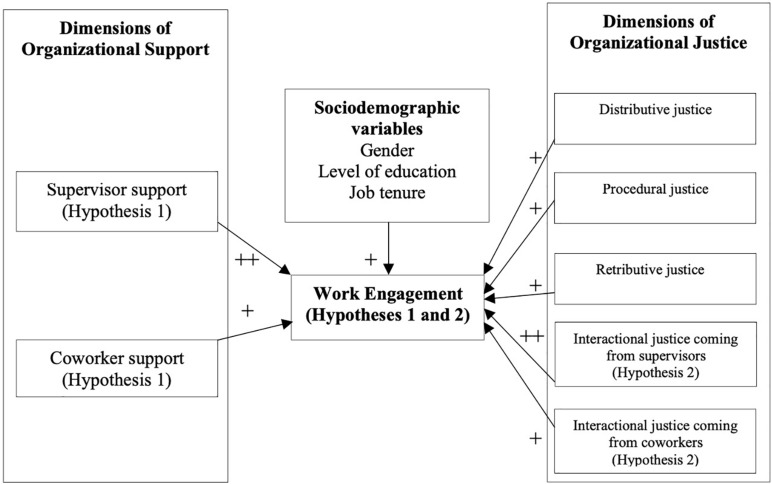
An overview of the conceptual model, hypotheses and variables used in the study.

As it has been found that employees’ WE may be affected by some demographic variables (Latta and Fait, 2016), we controlled for gender, level of education, and job tenure. Figure 1 gives an overview of the conceptual model, hypotheses and variables used in the study.

H1 and H2 in the figure refers respectively to Hypothesis 1: Supervisor support will be more strongly related to police officers’ work engagement than will coworker support and Hypothesis 2: Supervisor justice will be more strongly related with police officers’ work engagement than will coworker justice. The directions of arrows seen in the figure indicate how a variable influence another variable. The number of pluses (+) thus determines the strength of the influence, as in more pluses indicates a stronger influence.

### Data Analysis

Data were analyzed using SPSS 24.0. Means (M), standard deviations (SD), and Pearson’s correlation coefficient (r) were calculated. Correlation analysis was applied to test the direct relationships between OS, OJ, and WE, then we conducted a multiple regression analysis.

We also investigated the convergent and discriminant validity of our constructs. Convergent validity is the assessment to measure the level of correlation of multiple indicators of the same construct that are in agreement. To establish convergent validity, the factor loading of the indicator, Composite reliability (CR) and the Average Variance Extracted (AVE) have to be considered (Hair et al., 2013). CR was applied to test the degree to which the indicator variables converge and share proportion of variance. The CR value varies between 0 and 1; a higher value implies a higher level of reliability of the items (Hair et al., 2013). A cut-off point of 0.7 or above for CR is required to establish that the indicator items are reliable, and that they shared a high variance with the latent construct, although in exploratory studies the value of 0.6–0.7 is considered acceptable (Fornell and Larcker, 1981; Hair et al., 2010).

Discriminant validity, which confirms that the extent latent constructs are distinctly different (Houston, 2004; Zait and Bertea, 2011), was evaluated by examining AVE for each construct. This is established when the square root of AVE of each latent construct is greater than the construct’s highest correlation with other constructs in the model. We obtain discriminant validity if the AVE is greater than Maximum Shared Squared Variance (MSV) or Average Shared Squared Variance (ASV). For Convergent validity, AVE should be equal or greater than 0.50 and lower than CR. That is, the variance explained by the construct should be greater than measurement error and greater than cross-loadings (Rebelo-Pinto et al., 2014).

In addition, we employed the heterotrait-monotrait (HTMT) method, recently suggested by Henseler et al. (2015), to further check the degree to which the latent variables were distinctly different. The HTMT criterion is based on disattenuation correlation between two constructs.

The program AMOS was used to conduct a Structural Equation Model (SEM) in order to assess whether the data would fit the model.

Table 1 gives an overview of the different symbols and variables that were used in the present study.

**TABLE 1:** Overview of different variables, measures, abbreviations, and symbols used in the present study.

**Variables and measures**	**Abbreviations and symbols**
**Sociodemographic and control variables**	
Gender (0 = female, 1 = male)	–
Level of education	–
Job tenure	–
**Work engagement**	
Work engagement	WE
Vigor	–
Dedication	–
Absorption	–
Supervisor justice	SJ
Coworker justice	CWJ
**Dimensions of organizational support**	
Supervisor support	SS
Coworker support	CS
**Dimensions of Organizational justice**	
Distributive justice	DJ
Procedural justice	PJ
Retributive justice	RJ
Interactional justice coming from supervisors	–
Interactional justice coming from coworkers	–
**Hypotheses**	
Hypothesis 1	H1
Hypothesis 2	H2
Arrow with plus sign^a^	→^+^
Arrow with two plus signs^a^	→^+ +^
Means	M
Standard deviations	SD
Pearson’s correlation coefficient	r
**Regression analysis**	
Beta coefficient	β
R^2^ model change	Δ*R*^2^
Variance inflation factor	VIF
Asterisks that denote a strong partial correlation	*
**Reliability measure**	
Cronbach’s alpha coefficient	α_CR_
Structural equation modeling	SEM
**Measures of convergent and discriminant validity**	
Composite reliability	CR
Average variance extracted	AVE
Maximum shared variance	MSV
Average shared squared variance	ASV
Inter-scale correlations	–
Heterotrait-monotrait	HTMT

*– Indicates that no abbreviation and/or symbol was used for this particular variable or measure.*

*^a^The directions of arrows indicate how a variable influence another variable. The number of pluses (+) thus determines the strength of the influence, as in more pluses indicates a stronger influence.*

## Results

### Correlation Analysis

Table 2 presents the mean scores (M), standard deviations (SD), and correlations (Pearson’s r) for the study variables.

**TABLE 2:** Means (M), standard deviations (SD), and correlations (r) for study variables.

**Variable**	***M***	***SD***	**1**	**2**	**3**	**4**	**5**	**6**	**7**	**8**
1. SS	2.41	0.78	(0.92)							
2. CS	2.78	0.69	0.56	(0.91)						
3. DJ	1.96	0.80	0.54	0.26	(0.91)					
4. PJ	2.24	0.73	0.56	0.44	0.64	(0.83)				
5. RJ	2.59	0.73	0.51	0.37	0.38	0.49	(0.73)			
6. SJ	2.52	0.72	0.74	0.45	0.63	0.73	0.59	(0.88)		
7. CWJ	2.89	0.69	0.36	0.50	0.31	0.36	0.35	0.48	(0.73)	
8. WE	3.90	0.88	0.49	0.31	0.38	0.41	0.29	0.52	0.31	(0.93)

*N = 170. Cronbach’s alphas (α_*CR*_) are shown in parentheses on the diagonal. SS, supervisor support; CS, coworker support; DJ, distributive justice; PJ, procedural justice; RJ, retributive justice; SJ, supervisor justice; CWJ, coworker justice; WE, work engagement; r, Pearson’s correlation coefficient.*

Along with the increase in support (from superiors or colleagues) and organizational justice (of every dimension), work engagement grows. Support from colleagues is more closely related to work engagement than support from a supervisor. Likewise, the efficiency of colleagues is more related to work engagement than the efficiency of a supervisor. These results confirm both hypotheses 1 and 2. WE of police officers fell within the average range. It is interesting to note that support from colleagues was at a higher level than support coming from supervisors. The correlation analysis results showed that, out of all of the dimensions of OJ, supervisor justice had the strongest positive link with WE. Further, the significant positive correlation between WE and support from supervisors was stronger than that between WE and support from coworkers.

### Regression Analysis

The performed regression analysis shows that the support and justice of the superior has a greater impact on the work engagement of police officers than the support and efficiency of the employees. For a police officer on duty, the relationship with his superior influences the work engagement to a greater extent than the relationship with other police officers. The regression analysis results (see Table 3) show that the only significant predictors of WE were supervisor justice and supervisor support, with the effect of coworker support being non-significant. This particular model fitted the variables well, and OJ coupled with OS accounted for 26% of the variation in WE. Per the results shown in Tables 2, 3, both hypotheses were supported.

**TABLE 3:** Regression results for the effect of gender, level of education, job tenure, organizational support, and organizational justice on work engagement.

	**Variables**	**β**	**Δ*R*^2^**	**VIF**
Step 1	Controlled variables			
	Gender (0 = female, 1 = male)	−0.13	0.037	1.02
	Level of education	0.14		1.02
	Job tenure	−0.06		1.02
Step 2	Controlled variables			
	Gender (0 = female, 1 = male)	−0.10	0.260**	1.10
	Level of education	0.04		1.18
	Job tenure	−0.03		1.19
	**Independent variables**			
	Supervisor support	0.36*		2.26
	Coworker support	−0.11		2.12
	Distributive justice	0.07		2.00
	Procedural justice	0.06		3.00
	Retributive justice	0.02		1.80
	Interactional justice coming from supervisors	0.20**		2.41
	Interactional justice coming from coworkers	0.04		1.82

N = 170. β, beta coefficient; ΔR, R^2^*model change;* VIF, variance inflation factor. Asterisks * denote a strong partial correlation, *p < 0.05, **p < 0.01.

### Convergent and Discriminant Validity

The AVE represents the average amount of variance that a construct explains in its indicator variables relative to the overall variance of its indicators (Henseler et al., 2014). The AVE should be higher than any other variable correlation with the construct and > 0.50, known as the Fornell and Larcker criterion (Fornell and Larcker, 1981). However, recent research has revealed that the Fornell and Larcker criterion is not effective under certain circumstances (Rönkkö and Evermann, 2013; Henseler et al., 2014), pointing to a potential weakness in the most commonly used discriminant validity criterion. Malhotra and Dash (2011) argue that the AVE is often too strict, and reliability can be established through CR alone. AVE is a strict measure of convergent validity. They also note that “AVE is a more conservative measure than CR. On the basis of CR alone, the researcher may conclude that the convergent validity of the construct is adequate, even though more than 50% of the variance is due to error” (Malhotra and Dash, 2011, p. 702). Tables 4, 5 gives an overview over the Composite reliability (CR**),** the Average Variance Extracted (AVE), the Maximum Shared Variance (MSV) and inter-scale correlations for the variables used in the study.

**TABLE 4:** CR, AVE, MSV, and inter-scale correlations for study variables.

**Variable**	***CR***	***AVE***	**MSV**	**1**	**2**	**3**	**4**	**5**
1. SS	0.960	0.749	0.402	0.866				
2. CS	0.960	0.750	0.402	0.634	0.866			
3. DJ	0.958	0.767	0.441	0.876				
4. PJ	0.922	0.631	0.777	0.651	0.794			
5. RJ	0.033	0.370	0.896	0.625	0.882	0.608		
6. SJ	0.928	0.648	0.896	0.664	0.790	0.947	0.805	
7. CWJ	0.918	0.736	0.362	0.323	0.468	0.602	0.579	0.858
8. WE	0.925	0.429						

*N = 170. SS, supervisor support; CS, coworker support; DJ, distributive justice; PJ, procedural justice; RJ, retributive justice; SJ, supervisor justice; CWJ, coworker justice; WE, work engagement.*

**TABLE 5:** CR, AVE, MSV, and inter-scale correlations for study variables.

**Variable**	***CR***	***AVE***	**MSV**	**1**	**2**	**3**	**4**	**5**
**OS**								
1. SS	0.960	0.749	0.402	0.866				
2. CS	0.960	0.750	0.402	0.634	0.866			
**OJ**								
3. DJ	0.958	0.767	0.441	0.876				
4. PJ	0.922	0.631	0.777	0.651	0.794			
5. RJ	0.033	0.370	0.896	0.625	0.882	0.608		
6. SJ	0.928	0.648	0.896	0.664	0.790	0.947	0.805	
7. CWJ	0.918	0.736	0.362	0.323	0.468	0.602	0.579	0.858
**WE**								
8. Vigor	0.830	0.454	0.865	0.674				
9. Dedication	0.832	0.500	0.887	0.930	0.707			
10. Absorption	0.801	0.454	0.887	0.916	0.942	0.648		

*N = 170. OS, organizational support; SS, supervisor support; CS, *coworker support; OJ, organizational justice; DJ, distributive justice; PJ, procedural justice; RJ, retributive justice; SJ, supervisor justice; CWJ*, coworker justice; WE, work engagement.*

Looking at composite reliability (CR) values found in Tables 4, 5, they should be higher than of 0.70, as this cut-off point for CR is required to establish that the indicator items are reliable (Hair et al., 2013). All variables, except the variable retributive justice (RJ) exceeded this threshold, indicating that for these variables there was a high degree of convergent validity.

Also for convergent validity, AVE should be equal or greater than0.50 and lower than CR. As also can be seen in Tables 4, 5, AVE was greater than 0.50 for the variables supervisor support (SS), coworker support (CS), distributive justice (DJ), procedural justice (PJ), supervisor justice (SJ), coworker justice (CWJ), and dedication, indicating a high degree of discriminative validity for these variables. However, AVE was lower than 0.50 for WE (Table 4) and for vigor and absorption as variables of WE (Table 4), indicating a lack of discriminative validity for both WE and the constructs subscales. Also AVE was lower than 0.50 for RJ (Tables 4, 5) indicating a lack of discriminative validity for this variable. In addition, CR for WE in total was 0.925 but with an AVE less than 0.50, indicating a somewhat low degree of discriminative validity for this variable (Table 4).

Furthermore, taking a closer look at whether the average variance extracted (AVE) was greater than maximum shared squared variance (MSV), the picture changes slightly. AVE was higher than MSV for the variables SS, CS, DJ, CWJ, and dedication. AVE was on the other hand lower than MSV for the variables PJ, RJ, SJ, vigor and absorption indicating a lack of discriminant validity for these variables. For both the variables in organizational support (OS), that is SS and CS, AVE was higher than MSV, indicating a high degree of discriminant validity. Discriminant validity, which confirms the extent latent constructs are distinctly different (Houston, 2004; Zait and Bertea, 2011), was evaluated by examining AVE for each construct. This is established when the square root of AVE of each latent construct is greater than the construct’s highest correlation with other constructs in the model. We obtain discriminant validity if average variance extracted (AVE) is greater than maximum shared squared variance (MSV) or average shared squared variance (ASV). For convergent validity, AVE should be equal or greater than 0.50 and lower than CR. That is, variance explained by the construct should be greater than measurement error and greater than cross-loadings (Rebelo-Pinto et al., 2014).

Based on the before mentioned discussion and studies, we have therefore also included the heterotrait-monotrait (HTMT) ratio of correlations method, as this method has emerged as a valid method for establishing the discriminant validity assessment in variance-based Structural Equation Modeling (SEM). Thresholds for HTMT are 0.850 for strict and 0.900 for liberal discriminant validity (Henseler et al., 2015). When a HTMT value between two latent constructs is less than 0.850, discriminant validity is thus established according to Henseler et al. (2015). Table 6 gives an overview over the composite reliability (CR) and the heterotrait-monotrait (HTMT) analysis of the variables used in the study.

**TABLE 6:** CR and HTMT analysis for study variables.

		**HTMT analysis**			
**Variable**	***CR***	**1**	**2**	**3**	**4**
**OS**					
1. SS	0.960				
2. CS	0.960	0.634			
**OJ**					
3. DJ	0.958				
4. PJ	0.922	0.660			
5. RJ	0.033	0.302	0.457		
6. SJ	0.928	0.674	0.790	0.214	
7. CWJ	0.918	0.337	0.477	0.174	0.573
**WE**					
8. Vigor	0.830				
9. Dedication	0.832	0.849			
10. Absorption	0.801	**0.918**	**0.882**		

*N = 170. OS, organizational support; SS, supervisor support; CS, *coworker support; OJ, organizational justice; DJ, distributive justice; PJ, procedural justice; RJ, retributive justice; SJ, supervisor justice; CWJ*, *coworker justice; WE, work engagement.* The values (in bold) indicated discriminant validity problems.*

As can be seen from Table 6, CR values were found to be greater than 0.70, in line with recommendations (Fornell and Larcker, 1981; Hair et al., 2010), indicating a high degree of convergent validity. From the HTMT results, the values (in bold) indicated discriminant validity problems according to the strict HTMT 0.850 criterion for supervisor support (SS) and absorption and also according to the more liberal discriminant validity HTMT 0.900 criterion for coworker support (CS) and absorption. This implied that the HTMT criterion detected the collinearity problems among the latent constructs (multicollinearity) as indicated by Henseler et al. (2015). The construct of absorption thus suffers from collinearity problems. In other words, it contains the overlapping items from the respondents’ perception in the affected construct.

### Structural Equation Model in Order to Predict WE

In order to test our measurement model, we used the Structural Equation Modeling (SEM) method for WE prediction. This analysis helped us to eliminate weak items and reach a suitable measurement model (Marsh and Shavelson, 1985; Kats, 2013). However, because our sample was selected according to the our convenience, it is not a random sample, and neither probabilistic methods nor tests can be used. If dependencies are described using regression equations or SEM analysis, then we can only use those measures of goodness that indicate, for example, the amount of the explained variance, and not the probability of the model’s adequacy.

**FIGURE 2:**
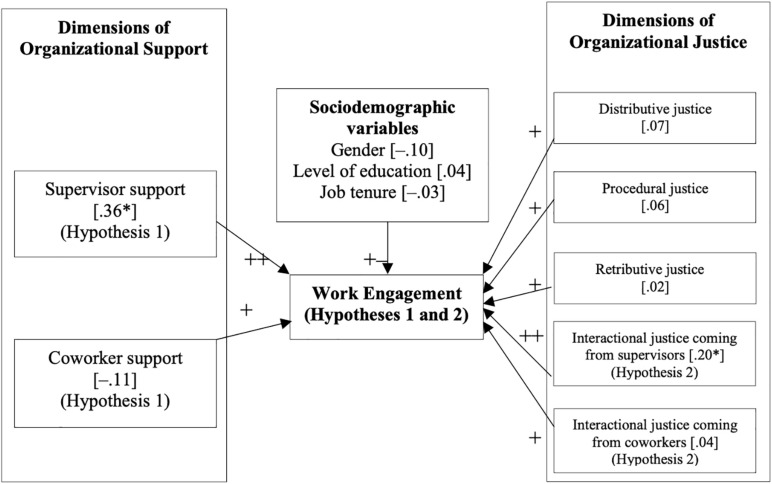
Structural equation model used for WE prediction. Asterisks ^∗^ denote a strong partial correlation. Pearson correlation coefficients are given in square brackets []. Asterisks ^∗^ denote and a statistically significant correlation (*p* = 0.05).

Figure 2 gives an overview of the SEM model and the share of the variance explained by the model, structural coefficients or regression coefficients.

As can be seen from the SEM model, when controlling for gender, level of education and job tenure, the effect of supervisor support upon WE was β = 0.36, lending support to our Hypothesis 1. Furthermore, the effect of interactional justice coming from supervisors, upon WE was β = 0.20, giving support to our Hypothesis 2.

## Discussion

Most of the existing studies on WE carried out among police officers have focused on the relationship between WE and OS, leaving out OJ almost entirely (Gillet et al., 2013).

WE was most strongly affected by supervisor support and supervisor justice, which highlights the fundamental role of one’s superiors in the forming of WE.

Our findings are particularly significant because few researchers have found results emphasizing the importance of support coming from superiors in the police force. Gillet et al. (2013) showed that the greater the organizational support is, the higher the police officers’ WE becomes. According to research by Gelderen and Bik (2016), conducted among policemen, supervisor support mediated the positive relationship between commitment, WE and extra-role performance.

According to Brunetto et al. (2017), proper OS in the police force can significantly reduce an officer’s intention to leave. Furthermore, Loi et al. (2006) argued that OJ, as mediated by perceived OS, leads to increased organizational commitment and decreased intention to leave. Jacobs et al. (2014) also claimed that OJ acting through OS increases ethical behaviors among police officers but in comparison to the aforementioned study, this study does not include OS as a mediator. Thus, it is important that police managerial staff take into account the importance of OJ and support if police officers are to be fully committed to their duty. A fair and supportive work environment enhances work engagement and protects policemen from burnout.

The present results suggest that changes are required not only in terms of the scope and content of leadership courses, but also in relation to the ways that police forces are managed. However, there is great resistance to systemic change in police forces, which results from the use of authoritarian management styles and motivation being predominantly driven by penalties (Rosado Diaz, 2015). The Polish police force, which is still resistant to implementing modern management methods, could potentially enhance its effectiveness and employee commitment if more attention was paid to the role of supervisors (Letkiewicz, 2012). For example, study programs in officer schools could have a greater emphasis on modern management concepts (Letkiewicz, 2007).

Composite reliability (CR) for WE in total was high but the Average Variance Extracted (AVE) was less than 0.50, indicating a somewhat low degree of discriminative validity. Furthermore, a lack of discriminant validity was yielded for two of the variables of WE, that is Vigor and Absorption. Also, the results from the heterotrait-monotrait (HTMT) method indicated discriminant validity problems for SS and absorption and for CS and absorption, implying that according to both the strict and more liberal HTMT criterion a collinearity problems among the latent constructs (multicollinearity) was detected.

## Conclusion

The purpose of our research was to investigate the relationships between OS, OJ, and WE in Polish police officers, and we found that positive relationships existed between these three variables.

Our main research question in this article was which of the dimensions of OJ and OS have the greatest relationship to WE among police officers. We have received an answer to this research question as both our two hypotheses, **Hypothesis 1:** Supervisor support will be more strongly related to police officers’ work engagement than will support from coworkers, and **Hypothesis 2:** Supervisor justice will be more strongly related with police officers’ work engagement than will coworker justice, were supported and confirmed.

The practical implication of this finding is that superiors should pay close attention to the fair and supportive treatment of their subordinates, and that training programs for leading staff and higher officers could be modified accordingly. We have filled a gap in the literature by conducting this study because, to the best of our knowledge, no similar studies regarding police officers exist.

Some limitations to this study should be noted. First, we did not include psychological variables as mediators in the relationships between OJ, OS, and WE. Future researchers should investigate the influence of psychological variables (e.g., conscientiousness and type A personality), combat mindset (Boe et al., 2020), resilience (Piotrowski et al., 2020b) and organizational variables (e.g., organizational climate and stress) on WE among Polish police officers. Second, prior researchers have found that OS mediates the relationship between OJ and police officers’ ethical behaviors. Thus, future researchers should examine the importance of OJ and support on police officers’ commitment to their ethical duties.

## Data Availability Statement

The raw data supporting the conclusions of this article will be made available by the authors, without undue reservation.

## Ethics Statement

The studies involving human participants were reviewed and approved by in accordance with the Code of Ethics and Professional Psychologist of the Polish Psychological Association (PTP). The patients/participants provided their written informed consent to participate in this study.

## Author Contributions

AP, SR, and OB contributed to conception and design of the study and wrote sections of the manuscript. AP organized the database, performed the statistical analysis, and wrote the first draft of the manuscript. All authors contributed to manuscript revision, read, and approved the submitted version.

## Conflict of Interest

The authors declare that the research was conducted in the absence of any commercial or financial relationships that could be construed as a potential conflict of interest.
